# Endovascular treatment of aortic coarctation using covered balloon-expandable stents—a systematic review and meta-analysis

**DOI:** 10.3389/fcvm.2024.1439458

**Published:** 2024-10-17

**Authors:** Fei He, Zhongze Cao, Chen Wang, Shyamal Premaratne, Benjamin W. Starnes, Chang Shu, Wayne W. Zhang

**Affiliations:** ^1^ Department of Vascular Surgery, Huaihe Hospital, Henan University, Kaifong, Henan, China; ^2^ Center of Vascular Surgery, Fuwai Hospital, Chinese Academy of Medical Sciences and Peking Union Medical College, Beijing, China; ^3^ Hunter Holmes McGuire Veterans Administration Medical Center, Richmond, VA, United States; ^4^ Virginia Union University, Richmond, VA, United States; ^5^ Division of Vascular and Endovascular Surgery, University of Washington, Seattle, WA, United States; ^6^ Department of Vascular Surgery, Second Xiangya Hospital of Central South University, Changsha, Hunan, China

**Keywords:** aortic coarctation, endovascular, stenting, congenital heart disease, hypertension

## Abstract

**Objectives:**

Balloon dilation followed by balloon-expandable stent implantation is an effective treatment for improving hemodynamic status in patients with coarctation of the aorta (CoA). However, limited evidence exists regarding the safety and efficacy of covered balloon-expandable stents (CBSs) in a large cohort. In this meta-analysis, we aimed to evaluate the overall success rates, hemodynamic and anatomical benefits, complications, and mid-term results of CBSs in treating CoA.

**Methods:**

The PubMed, Embase, and Cochrane Library databases were systemically searched for studies reporting outcomes of CBSs in treating CoA. Single-group rate meta-analyses were performed to calculate estimated pooled procedural success rates, the incidence of complications, and re-coarctation rates. A meta-analysis using standardized mean differences was conducted to compare pre- and postoperative trans-coarctation pressure gradients (PGs), coarctation diameter, and overall changes in systolic blood pressure (SBP). Subgroup analyses were performed to identify potential sources of heterogeneity.

**Results:**

The final analysis included 12 studies with a total of 411 patients. The estimated pooled procedural success rate was 100% [95% confidence interval (CI): 98%–100%, *I*2 = 0, *P* = 0.78]. Significant decreases in trans-coarctation PGs and SBP were observed. The pooled incidences of stent-related, aortic, and access site complications were 2% (95% CI: 0%–5%, *I*^2^ = 30.4%, *P* = 0.15), 2% (95% CI: 0%–4%, *I*^2^ = 0%, *P* = 0.76), and 3% (95% CI: 1%–7%, *I*^2^ = 52.9%, *P* = 0.02), respectively. Subgroup analyses showed that implantation of BeGraft stents was related to a significantly higher incidence of access site complications.

**Conclusion:**

Covered balloon-expandable stent implantation in treating CoA is safe and effective with high procedural success rates, an acceptable incidence of complications, and a low incidence of re-coarctation.

**Systematic Review Registration:**

https://www.crd.york.ac.uk/PROSPERO/, PROSPERO (CRD42023430356).

## Introduction

Coarctation of the aorta (CoA) has a mean incidence of 409 per million live births and accounts for 5%–8% of all cases of congenital heart disease (1, 2). Depending on the extent and location of the coarctation, the clinical presentation of CoA includes upper extremity hypertension and lower body hypoperfusion (3). Untreated CoA may eventually lead to various complications such as uncontrollable systemic hypertension, congestive heart failure, and hemorrhagic stroke (3). The reported mean age of death for untreated CoA patients is 34 years (4).

Early intervention following the diagnosis of CoA is recommended. The goal of treatment is to eliminate the aortic pressure gradient (PG) or at least reduce systemic hypertension (3). Although surgical repair of the coarctated segment remains the treatment of choice, particularly for patients early in their lives, endovascular treatments, such as balloon dilation and bare stent implantation, have recently emerged as less invasive alternatives to surgery (5). However, bare stent implantation is associated with fatal complications, including aortic rupture, pseudoaneurysm formation, post-implantation aneurysm formation, and, in some cases, death (6, 7).

In adult CoA patients, dilation and stenting of the coarcted aorta entail a significant risk of aortic wall injury. Covered balloon-expandable stents (CBSs) have been utilized as a less invasive approach to mitigate treatment-related complications (8). CBSs have also been used to address complicated CoA lesions associated with severe aortic tortuosity, aneurysms, irregular aortic walls, and patent ductus arteriosus (PDA) (9–11). The current literature on CBS implantation in treating CoA is limited, with most of the evidence based on retrospective cohort studies. The primary purpose of this study was to systemically elucidate the safety and efficacy of CBSs in treating CoA.

## Methods

This meta-analysis was conducted in accordance with principles elaborated in the Preferred Reporting Items for Systematic Reviews and Meta-Analyses (PRISMA) statement (12). This study is also registered with PROSPERO (ID: CRD42023430356).

### Literature search strategy

We searched the PubMed, Embase, and Cochrane Library databases using the following search strategy: “coarctation* OR coarctated OR coarctate OR re-coarctation* OR re-coarctation*” AND “stent OR stents OR stenting OR stented OR percutaneous OR catheter* OR transcatheter OR trans catheter OR transluminal OR endovascular OR intravascular OR transvascular OR angioplast* OR TEVAR OR thoracic endovascular aortic repair OR endovascular stent grafting OR endovascular aortic repairs”. Exact search terms are summarized in Supplementary Material S1. A search for unpublished materials was not conducted.

### Study selection

**Figure 1:**
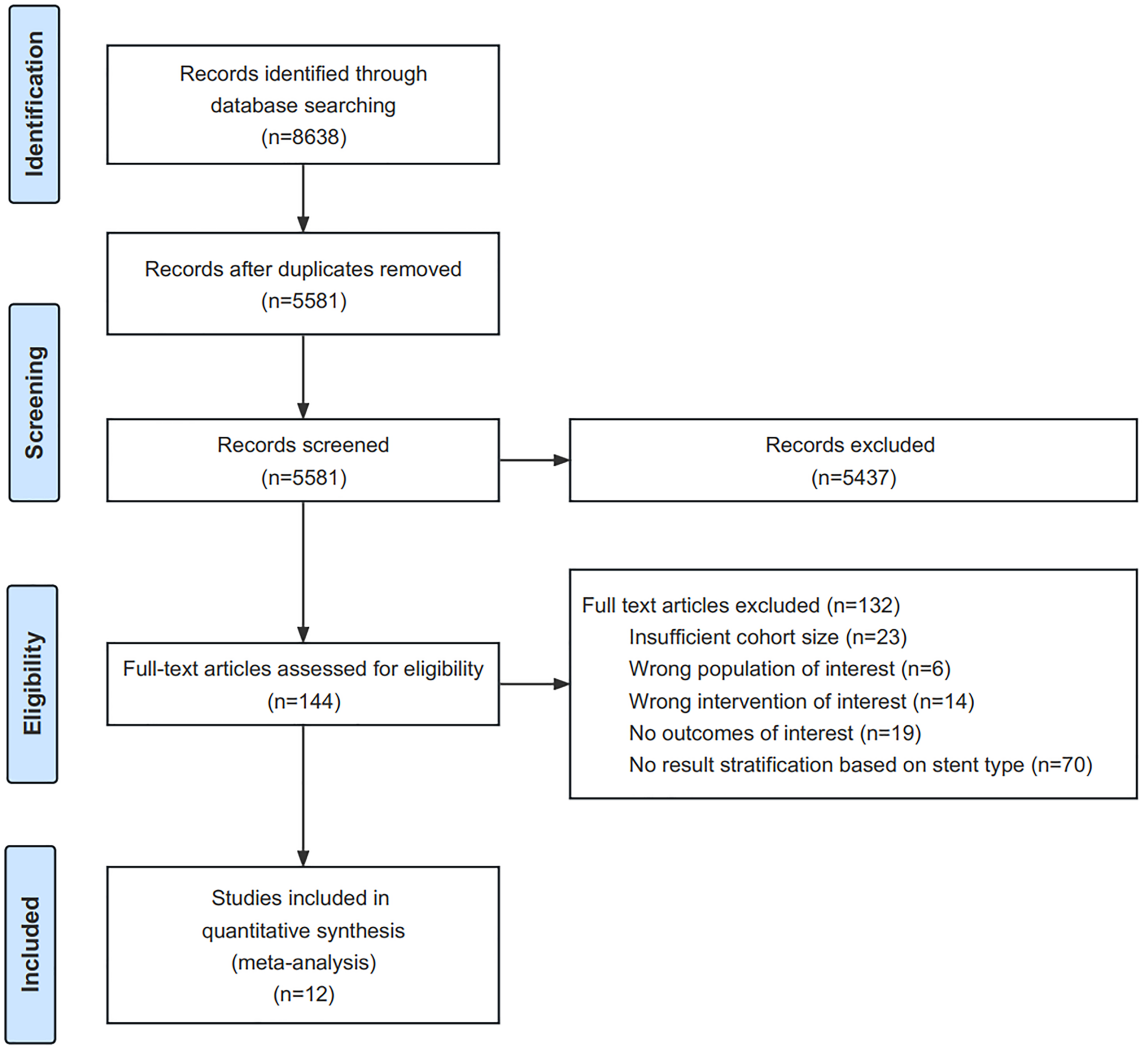
Step-by-step flowchart of the final analysis that included 12 studies (*n* = 12).

All identified papers published online prior to 31 March 2023 were included in this review. A total of 5,581 papers were identified. We excluded reviews, case reports, comments, letters to the editor, animal studies, non-English articles, studies including only infant patients, and reports irrelevant to CoA during screening. Full-text article assessments were conducted on 144 articles. Studies meeting the following criteria were included: (1) those including at least 10 patients; (2) those reporting stratified outcomes of treating native or recurrent CoA with covered balloon-expandable stents; (3) those reporting at least one of the following outcomes: technical success, trans-coarctation pressure gradient before and after stent implantation, perioperative complications, and complications during follow-up. The final analysis included 12 studies (Figure 1).

### Quality appraisal and data extraction

The quality of the included studies was reviewed independently by the first two authors using the Joanna Briggs Institute (JBI) Checklist for Case Series (13). The checklist assessed the criteria and completeness of inclusion, measurement validity, reporting of demographics and clinical information, outcomes and follow-up results, and statistical analysis methods. The available responses for items in the checklist included “Yes,” “No,” “Unclear,” and “Not applicable.” Studies receiving >1 “No” or >3 “Unclear” responses were excluded from the final analysis to limit the risk of bias. Any discrepancies during the assessment were resolved by discussion among all authors.

Data from the included studies were extracted and entered into an electronic database. The database was reviewed and confirmed by all authors. Associated abnormalities recorded were aneurysms, PDA, and aortic arch hypoplasia, which is a congenital heart defect where the arch is too narrow. Intra-cardiac congenital abnormalities were considered irrelevant to stent implantation and were not included during data extraction. Complications occurring immediately after the procedure and during follow-up were recorded.

Although one included study strictly defined success as a systolic gradient of <10 mmHg from the ascending to descending aorta without aortic wall injury (14), in this analysis, procedural success was defined as an immediate reduction in the trans-coarctation pressure gradient to less than 20 mmHg and the exclusion of associated lesions after the initial procedure. Planned further dilation is described as staged balloon dilation of the CBS, with/without additional bare stent implantation. Stent-related complications included tears in the stent covering, stent strut fractures, stent collapse, and stent migration. Aortic complications included post-stenting aortic dissection, pseudoaneurysm formation, aneurysm formation, and aortic kinking proximal to the stent. Access complications included pseudoaneurysm or hematoma formation at the access site, as well as iatrogenic stenosis or occlusion of the access vessel. Re-coarctation after stenting was defined as a pressure gradient of ≥20 mmHg across the stented area during follow-up, lumen loss of >30% based on computed tomography angiography (CTA) measurements, or the reporting of re-coarctation/restenosis in the included publications.

### Statistical analysis

All analyses were performed using Stata version 15.1 (StataCorp, College Station, TX, USA). Median and quartiles were transformed into the mean and standard deviation (SD) format to facilitate analysis. A *P-*value of *<*0.05 for Cochran's *Q* statistic or an *I*^2^ >50% indicated a significant difference. Confidence intervals (CIs) below zero were reported as zero. Dichotomous outcomes were pooled using the Freeman–Tukey double arcsine transformation to improve the normality of data distribution. Heterogeneity was assessed using the inverse-variance random-effects model. The method of DerSimonian and Laird was applied for random-effects meta-analysis.

For continuous variables, standardized mean differences (SMDs) were generated using Cohen’s method to evaluate treatment efficacy. CIs were generated using the random-effects model or the fixed-effects model when heterogeneity was significant or insignificant, respectively. The results were presented as SMDs with 95% CIs. Reporting bias was not assessed in this analysis due to the lack of a suitable tool for evaluating reporting bias in a meta-analysis of pooled proportions and incidences. Subgroup analyses based on sample size, mean age, proportion of native CoA, stent type, and follow-up duration were conducted to explore potential sources of heterogeneity.

## Results

### Study characteristics

The analysis included 10 retrospective studies, 1 prospective study, and 1 randomized controlled trial (Table 1). The quality assessment of all included studies is presented in Supplementary Table S1. All patients were diagnosed with aortic CoA and underwent endovascular CoA treatment using CBSs. The stents used in the studies included the covered Cheatham Platinum (CCP) stent (Numed Inc., Hopkinton, NY, USA), Advanta V12™ stent (Atrium Medical Corp., Hudson, NH, USA), and BeGraft covered stent (Bentley Innomed, Hechingen, Germany).

**Table 1:** Characteristics of included studies.

Author (year)	Sample size	Stent type	Age (years)	Male	Native coarctation	Aneurysm	PDA	Arch hypoplasia	Planned further dilation	FU (m)
Tzifa (2006) (9)	30	CCP	28.0	NA	14	8	2	NA	4	11.0
Butera (2007) (10)	33	CCP	18.2	23	20	10	1	NA	0	13.9
Bruckheimer (2009) (11)	22	CCP	17.7	14	22	NA	NA	NA	9	17.9
Tanous (2010) (15)	22	CCP	39.0	11	14	5	0	1	0	12.0
Chang (2012) (16)	25	CCP	22.5	16	25	NA	5	2	0	32.0
Ohno (2013) (17)	17	Advanta	12.9	14	13	NA	NA	7	1	9.6
Sohrabi (2014) (8)	60	CCP	22.5	40	60	NA	NA	0	0	31.1
Promphan (2020) (18)	12	BeGraft	19.9	8	8	NA	2	2	2	10.3
Stassen (2020) (20)	89	CCP	23.9	60	57	NA	NA	NA	22	79.2
Sasikumar (2020) (14)	20	Advanta (*n* = 13) CCP (*n* = 7)	34.8	13	16	NA	NA	NA	4	62.4
Yilmazer (2021) (21)	11	BeGraft	15.2	7	5	NA	1	0	0	14.2
Bruckheimer (2021) (19)	70	Advanta	20.3	43	55	NA	NA	NA	16	NA
Pooled results (95% CI)	411	CCP (*n* = 288) Advanta (*n* = 100) BeGraft (*n* = 23)	22.4 (19.0–25.7)^c^	66 (61–71)^b^	79 (64–91)^b^	27 (18–37)^b^	7 (1–15)^b^	7 (0–22)^b^	8 (2–28)^b^	25.4 (19.3–31.5)^c^

CCP, covered Cheatham Platinum stent; FU, follow-up; PDA, patent ductus arteriosus; RCT, randomized controlled trial; NA, not accessible; CI, confidence interval.

^a^ Pooled counts (95% CI).

^b^ Pooled percentages (95% CI).

^c^ Pooled mean (95% CI).

### Patient demographics

The final analysis included 411 patients. The pooled estimate of age was 22.4 years (95% CI: 19.0–25.7, *I*^2^ = 91.8%, *P* < 0.001). The estimated pooled proportion of patients with native CoA from 12 studies was 79% (95% CI: 64%–91%, *I*^2^ = 89.8%, *P* < 0.001) (8–11, 14–21). A total of 23 cases complicated by thoracic aortic aneurysms were reported in three studies (9, 10, 15), with an estimated concurrence of 27% (95% CI: 18%–37%, *I*^2^ = 0, *P* = 0.85). A total of 11 cases complicated by a PDA were reported in six studies (9, 10, 15, 16, 18, 21), with an estimated concurrence of 7% (95% CI: 1%–15%, *I*^2^ = 44.8%, *P* = 0.11). A total of 12 cases complicated by aortic arch hypoplasia were reported in six studies (8, 15–18, 21), with an estimated concurrence of 7% (95% CI: 0%–22%, *I*^2^ = 80.5%, *P* < 0.001). The rate of planned further dilation from all included studies was estimated to be 8% (95% CI: 8%–28%, *I*^2^ = 85.0%, *P* < 0.001). The characteristics of the included studies are summarized in Table 1.

### Efficacy of the treatment

**Figure 2:**
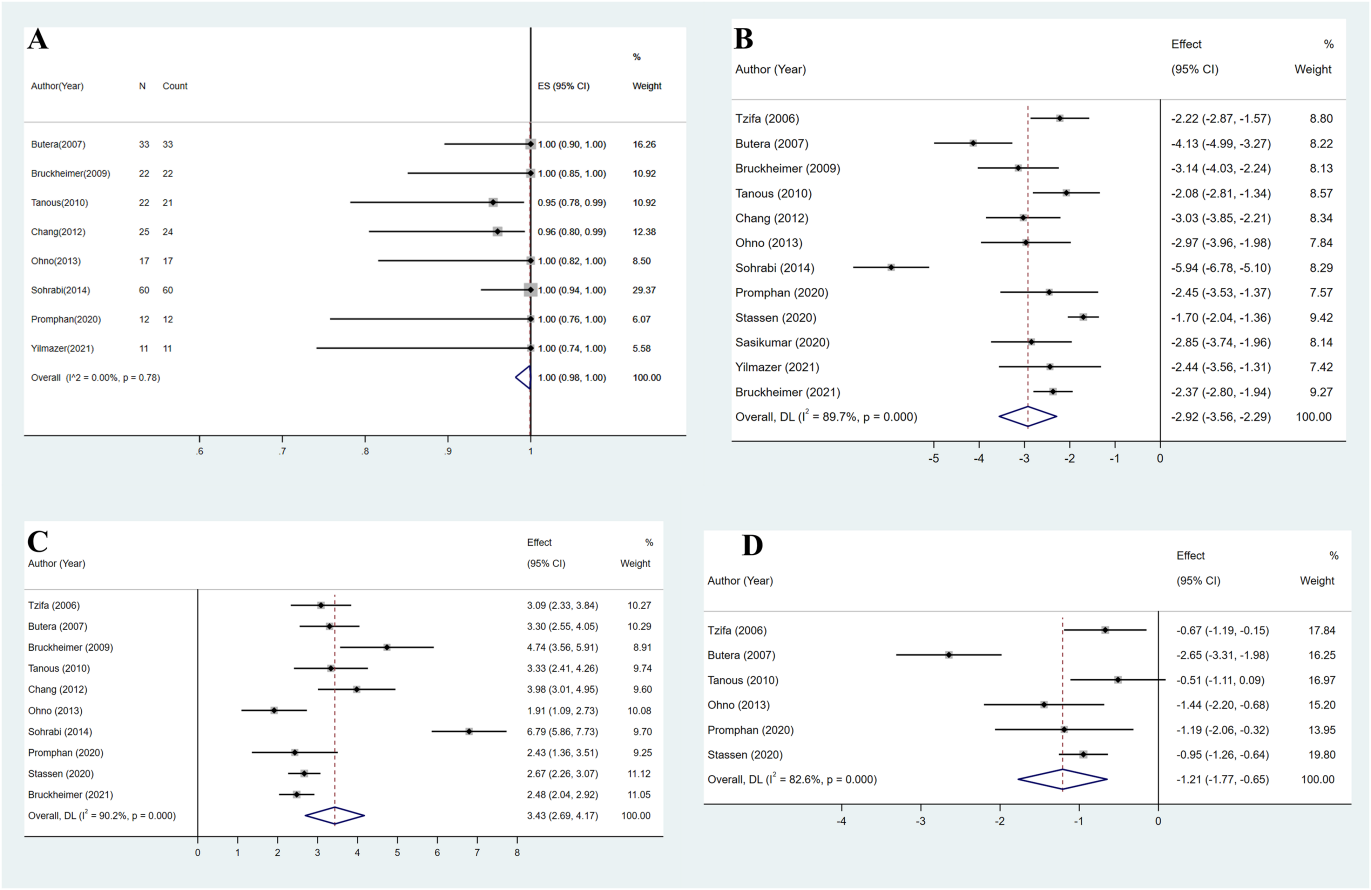
**(A)** Overall estimated pooled procedural success. **(B)** Overall estimated pooled pressure gradient change. **(C)** Overall estimated pooled coarctation diameter change. **(D)** Overall estimated pooled systolic blood pressure change.

The estimated pooled technical success rate from eight studies was 100% (95% CI: 98%–100%, *I*^2^ = 0, *P* = 0.78) (Figure 2A) (8, 10, 11, 15–18, 21). The remaining studies were excluded due to the lack of postprocedural trans-coarctation PG maximum or individual PG values (9, 14, 19, 20). All included studies recorded trans-coarctation PG values before and after the procedure (8–11, 14–21). After CBS implantation, the estimated pooled mean PG decreased from 38.0 mmHg (95% CI: 30.7–45.3 mmHg, *I*^2^ = 96.0%, *P* < 0.001) before the procedure to 3.6 mmHg (95% CI: 2.7–4.5 mmHg, *I*^2^ = 77.4%, *P* < 0.001). On average, the trans-coarctation PG was 2.9 SDs (95% CI: 2.3–3.6, *I*^2^ = 89.7%, *P* < 0.001) lower after CBS implantation compared with the baseline PG (Figure 2B).

The pooled mean minimum diameter of the coarctated aorta from 10 studies increased from 5.9 mm (95% CI: 4.5–7.4 mm, *I*^2^ = 97.5%, *P* < 0.001) before the procedure to 15.4 mm (95% CI: 13.9–16.8 mm, *I*^2^ = 96.0%, *P* < 0.001) after CBS implantation (8–11, 15–20). On average, CBS implantation increased the minimum diameter of the coarctated aorta by 3.4 SDs (95% CI: 2.7–4.2, *I*^2^ = 90.2%, *P* < 0.001) (Figure 2C).

Systolic blood pressure (SBP) data were available from six studies (9, 10, 15, 17, 18, 20). SBP was measured by cuff at rest on the left arm in one study (17) and on the right arm in two studies (15, 20), while three studies did not provide further details (9, 10, 18). After pooling, the estimated mean SBP decreased from 145.7 mmHg (95% CI: 138.9–152.5 mmHg, *I*^2^ = 83.2%, *P* < 0.001) preoperatively to 125.2 mmHg (95% CI: 118.4–132.0 mmHg, *I*^2^ = 89.3%, *P* < 0.001) during follow-up. On average, mean SBP during follow-up was 1.2 SDs (95% CI: 0.7–1.8, *I*^2^ = 82.6%, *P* < 0.001) lower compared with preoperative SBP (Figure 2D).

### Complications

**Figure 3:**
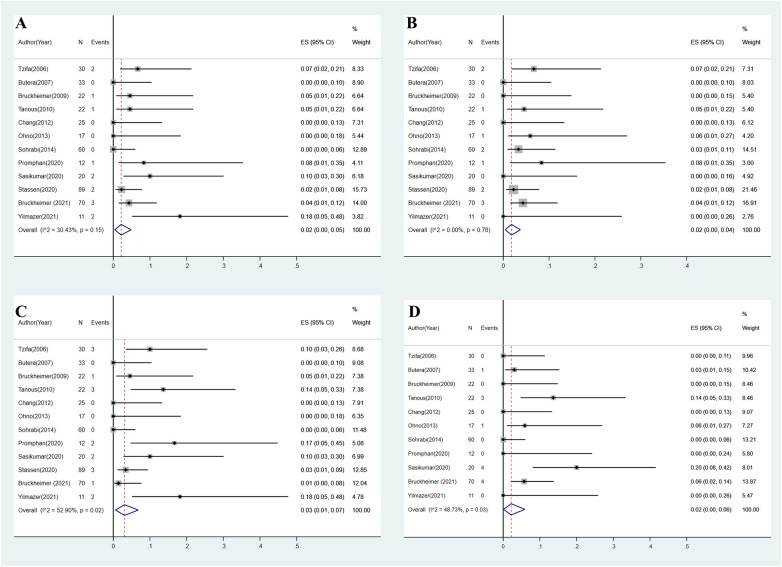
**(A)** Overall estimated pooled incidence of stent-related complications. **(B)** Overall estimated pooled incidence of aortic complications. **(C)** Overall estimated pooled incidence of access site complications. **(D)** Overall estimated pooled incidence of re-coarctation.

A total of 14 stent-related complications were reported across all included studies (8–11, 14–21). Reported complications included stent fracture (*n* = 5) (9, 15, 20), stent strut distortion (*n* = 2) (18, 21), stent infolding or collapse (*n* = 5) (14, 19), stent covering tear (*n* = 1) (11), and stent migration (*n* = 1) (21). All stent fractures were reported after CCP stent implantation. Stent infolding and collapse were observed only in Advanta stents. Strut distortion and stent migration were observed only after BeGraft stent implantation. The overall estimated pooled incidence of stent-related complications was 2% (95% CI: 0%–5%, *I*^2^ = 30.4%, *P* = 0.15) (Figure 3A).

Aortic complications were reported in 12 patients across all included studies (8–11, 14–21), including aortic pseudoaneurysm (*n* = 4) (8, 15, 18), aneurysm formation (*n* = 3) (19, 20), aortic dissection (*n* = 2) (9, 19), aortic hematoma (*n* = 1) (19), and kinking of the aorta proximal to the stent (*n* = 2) (9, 17). The majority of complications were observed after CCP stent implantation (*n* = 7). The overall estimated pooled incidence of aortic complications was 2% (95% CI: 0%–5%, *I*^2^ = 0%, *P* = 0.76) (Figure 3B).

As the most frequent complication, access site complication was observed in 17 patients across all included studies (8–11, 14–21). Reported access site complications included access site hematoma or bleeding (*n* = 4) (9, 18, 20), femoral pseudoaneurysm (*n* = 2) (11, 20), femoral artery aneurysm formation (*n* = 1) (9), access vessel thrombosis or occlusion (*n* = 5) (15, 19–21), iliac artery stenosis (*n* = 2) (15, 21), pedal pulse reduction (*n* = 1) (18), and unknown complications (*n* = 2) (14). The overall estimated pooled incidence of access site complications was 3% (95% CI: 1%–7%, *I*^2^ = 52.9%, *P* = 0.02) (Figure 3C).

Data on re-coarctation after CBS implantation were available from 11 studies (8–11, 14–19, 21). A total of 13 re-coarctation cases were reported. Criteria for re-coarctation diagnosis included stenosis from endothelial proliferation (*n* = 2) (10, 17), trans-coarctation PG >20 mmHg during follow-up angiography (*n* = 3) (15), lumen loss >30% based on radiologic follow-up (*n* = 4) (14), and unreported criteria (*n* = 4) (19). The overall estimated pooled incidence of re-coarctation was 2% (95% CI: 0%–6%, *I*^2^ = 48.7%, *P* = 0.03) (Figure 3D).

### Subgroup analysis

Tables 2, 3 summarize the stratification of studies by various subgroups and the relevant results. The influence of mean patient age, proportion of native CoA, and stent type on procedural success and trans-coarctation pressure gradient change was not significant. However, the overall decrease in SBP was significantly greater in studies including patients with a mean age <20 years.

**Table 2:** Procedural success, pressure gradient changes, and systolic blood pressure changes among different subgroups.

Subgroup	Procedural success				Pressure gradient change				Systolic blood pressure change			
	Studies	*I*^2^ (%)	Proportion, 95 CI% (%)	*P*-value	Studies	*I*^2^ (%)	ES (95% CI)	*P*-value	Studies	*I*^2^ (%)	ES (95% CI)	*P*-value
Sample size	0.95				0.51				0.74			
>20 patients	5	0	100 (97–100)	0.41	8	93.3	−3.04 (−3.89 to −2.19)	<0.001	4	89.1	−1.17 (−1.93 to −0.41)	<0.001
≤20 patients	3	—	100 (98–100)	—	4	0	−2.71 (−3.22 to −2.21)	0.85	2	0	−1.34 (−1.91 to −0.76)	0.67
Mean age (years)	0.52				0.67				**0.04**			
≥20	3	—	99 (93–100)	—	7	93.2	−2.85 (−3.72 to −1.98)	<0.001	3	0	−0.82 (−1.06 to −0.57)	0.37
<20	5	0	100 (98–100)	1.00	5	51.4	−3.08 (−3.71 to −2.45)	0.08	3	77.1	−1.79 (−2.71 to −0.87)	0.01
Native CoA	0.62				0.15				0.59			
<75% native CoA	4	0	100 (96–100)	0.67	6	81.7	−2.46 (−3.14 to −1.77)	<0.001	5	85.6	−1.17 (−1.82 to −0.52)	<0.001
≥75% native CoA	4	0	100 (98–100)	0.53	6	91.0	−3.37 (−4.41 to −2.33)	<0.001	1	\	−1.45 (−2.20 to −0.69)	\
Stent type	0.99				0.52				0.86			
CCP	5	0	100 (97–100)	0.41	7	94.3	−3.15 (−4.22 to −2.08)	<0.001	4	89.1	−1.17 (−1.93 to −0.41)	<0.001
Advanta	1	—	100 (80–100)	—	2	15.1	−2.50 (−2.98 to −2.02)	0.28	1	\	−1.45 (−2.20 to −0.69)	\
BeGraft	2	—	100 (92–100)	—	2	0	−2.45 (−3.23 to −1.67)	0.98	1	\	−1.19 (−2.06 to −0.32)	\
Follow-up duration (months)	0.37				0.10				0.31			
>12	5	0	100 (98–100)	0.67	8	93.2	−3.18 (−4.09 to −2.27)	<0.001	2	95.1	−1.77 (−3.44 to −0.11)	<0.001
≤12	3	—	99 (93–100)	—	4	0	−2.33 (−2.74 to −1.93)	0.53	4	34.9	−0.88 (−1.29 to −0.46)	0.20

CCP, covered Cheatham Platinum stent; CoA, coarctation of the aorta; CI, confidence interval; ES, effect size.

Bold values indicate a statistically significant difference between subgroups.

**Table 3:** Comparison of incidences of complications and re-coarctation between different subgroups.

Subgroup	Stent-related complications				Aortic complications				Access site complications				Re-coarctation			
	Studies	*I*^2^ (%)	Proportion, 95 CI% (%)	*P*-value	Studies	*I*^2^ (%)	Proportion, 95 CI% (%)	*P*-value	Studies	*I*^2^ (%)	Proportion, 95 CI% (%)	*P*-value	Studies	*I* ^2^	Proportion, 95 CI% (%)	*P*-value
Cohort size	0.08				0.66				0.09				0.24			
>20 patients	8	11.7	2 (0–4)	0.34	8	0	2 (1–4)	0.67	8	48.6	2 (0–5)	0.06	7	47.1	2 (0–5)	0.08
≤20 patients	4	24.0	7 (0–17)	0.27	4	0	2 (0–9)	0.51	4	37.8	8 (1–21)	0.19	4	40.5	2 (0–6)	0.17
Mean age (years)	0.62				0.63				0.64				0.65			
≥20	7	31.6	2 (0–5)	0.19	7	0	2 (1–5)	0.76	7	57.7	3 (0–7)	0.03	6	72	3 (0–10)	<0.001
<20	5	41.6	3 (0–10)	0.14	5	0	1 (0–5)	0.43	5	54.7	4 (0–13)	0.07	5	0	1 (0–5)	0.81
Native CoA	0.41				0.90				0.05				0.96			
<75%	6	34.7	3 (0–8)	0.18	6	0	2 (0–5)	0.49	6	58.0	6 (1–14)	0.04	5	25.7	2 (0–7)	0.25
≥75%	6	33.6	2 (0–5)	0.18	6	0	2 (0–5)	0.69	6	21.9	1 (0–4)	0.27	6	64.5	3 (0–9)	0.01
Stent type	0.09				0.59				**0.02**				**0**.**03**			
CCP	7	33.3	2 (0–4)	0.17	7	0	2 (0–4)	0.62	7	54.7	2 (0–7)	0.04	6	42.4	1 (0–4)	0.12
Advanta	1	—	0 (0–2)	—	2	—	4 (0–10)	—	2	—	1 (0–4)	—	2	—	5 (1–11)	—
BeGraft	2	—	1 (1–3)	—	2	—	3 (0–16)	—	2	—	17 (4–37)	—	2	—	0 (0–8)	—
Follow-up duration (months)	0.32				**0**.**04**				0.05				0.67			
>12	8	43.9	2 (0–5)	0.09	8	0	1 (0–3)	0.87	8	42.4	2 (0–5)	0.10	7	55.6	2 (0–7)	0.04
≤12	4	0	4 (0–10)	0.62	4	0	6 (1–13)	0.97	4	30.5	8 (2–18)	0.23	4	46.5	3 (0–12)	0.13

CCP, covered Cheatham Platinum stent; CoA, coarctation of the aorta; CI, confidence interval; ES, effect size.

Bold values indicate a statistically significant difference between subgroups.

In addition, the influence of sample size, mean age, and proportion of native CoA on postoperative complications and re-coarctation was not significant. Postoperative stent-related complications and aortic complications were not significantly related to stent types. However, the use of the BeGraft stent was associated with a significantly higher prevalence of access site complications. The association between Advanta stent implantation and a higher incidence of re-coarctation during follow-up was significant. Aortic complications were also observed in a significantly greater proportion of patients in studies with a mean follow-up duration of ≤12 months.

## Discussion

In this meta-analysis, significant decreases in trans-coarctation PG and SBP and a significant increase in coarctation diameter were observed after the implantation of all three types of CBSs. The estimated pooled incidences of stent-related, aortic, and access site complications were 2% (95% CI: 0%–5%, *I*^2^ = 30.4%, *P* = 0.15), 2% (95% CI: 0%–4%, *I*^2^ = 0%, *P* = 0.76), and 3% (95% CI: 1%–7%, *I*^2^ = 52.9%, *P* = 0.02), respectively.

### Procedural success and outcomes

Compared with patients receiving bare balloon-expandable stent implantation, those receiving CBSs are generally complicated by challenging CoA lesions associated with severe aortic tortuosity, aneurysms adjacent to the CoA, proximity to the left subclavian artery, or proximity with a PDA (9–11). CBS implantation is also used as a bail-out strategy for intraoperative aortic or stent-related complications (8). The differences between bare and covered stents are important. Covered stents are removable but can migrate and potentially cause cholecystitis if they obstruct the cystic duct. On the other hand, uncovered stents have a lower risk of causing cholecystitis but a higher rate of tissue ingrowth, and they are also removable.

Considering the unique demographics of patients receiving CBS implantation, procedural success in this study was defined as a decrease in the trans-coarctation pressure gradient to less than 20 mmHg and the exclusion of associated aneurysms or PDA after the initial procedure. In some cases, planned further dilation of CoA to transverse the arch diameter is scheduled after stenting. If the postprocedural PG meets the aforementioned criteria, the procedure is still considered successful.

All complications occurring immediately following stent implantation, following reintervention, and during follow-up were recorded. Due to the low prevalence of complications, no further stratified analysis was conducted based on the time between CBS implantation and complication occurrence. All CBS-related stent fractures were reported only after CCP stent implantation (9, 15, 20). Therefore, care must be taken for appropriate balloon diameter and pressure during dilation. In addition, CCP stents are hand-crimped onto the balloon during the operation, and inappropriate crimping torsion and diameter may lead to structural damage to the stent. Stent infolding and collapse were observed only after Advanta stent implantation (18, 19, 21). In 2018, a study analyzed the efficacy of self-expandable thoracic aortic endografts in treating CoA. The study reported a technical success rate of 100%, with no 30-day mortality or paraplegia events. One-year freedom from reintervention was 78% ± 9% (95% CI, 42%–92%) (22). However, the cohort included only 21 patients, and further evidence is required to assess the capability of cobalt-chromium endografts to resist the recoil force induced by CoA.

In cases complicated by excessive tortuosity or a modest CoA diameter, both proximal and distal anchoring portions of the stent may be exposed to excessive radial resistance. The biomechanical interaction between stenting grafts and the aorta in the setting of CoA still requires further investigation to optimize the results of endovascular CoA treatment. In addition, strut distortion and stent migration were observed only after BeGraft stent implantation, which used 316L stainless steel for the struts (21). It has been reported that distortion may arise from balloon retrieval, underlining the necessity for careful manipulation after stent implantation and improvements in the performance of existing delivery systems.

In particular, aortic kinking proximal to the CBS after implantation was considered an aortic complication in this analysis. The etiology of this complication may be attributed to increased vascular resistance and reduced compliance of aortic segments adjacent to CoA. Since reports on this phenomenon are scarce, vascular kinking after stent implantation may represent a new type of complication that requires further investigation. In this analysis, we also observed that aortic complications after CBS implantation were not rare (8, 15, 18). Although CBS implantation was initially utilized as a bail-out strategy to address potential aortic wall injuries after bare metal stent implantation in treating CoA, it can still cause aortic complications if implanted indiscreetly. The optimal diameter ratio between dilated CBS and CoA still requires further investigation.

### Subgroup analysis

Based on the results of the subgroup analysis (attached), the incidence of access site complications was significantly higher after BeGraft implantation (*P* = 0.02). A potential explanation may be the differing designs of delivery systems and methods. Before implantation, CCP stents are hand-crimped onto balloons and can result in smaller stent diameters introduced through 9–10-Fr sheaths (11). In comparison, Advanta stents are introduced through 8- or 11-Fr sheaths, while BeGraft stents are introduced through 9–14-Fr sheaths (17, 21). The larger caliber of the BeGraft delivery system may have contributed to increased access complication rates. Although the majority of access site complications are resolved without further treatment, it is important to implement careful access evaluation since CoA patients tend to be younger, and iatrogenic trauma of lower extremity vessels may exert negative effects on physical development.

Another important observation from subgroup analyses is that the overall decline in SBP was significantly more pronounced in patients younger than 20 years compared with those aged 20 years and older [effect size (ES) = −1.79, 95% CI = −2.71 to −0.87 vs. ES = −0.82, 95% CI = −1.06 to −0.57, *P* = 0.045]. This result contradicts a previous meta-analysis reporting a greater SBP decrease in patients aged 18 years and older (23). Our explanations for the result are as follows: (i) adult patients receiving CBSs are generally older, and maladaptive vascular change from persisting CoA may contribute to an insufficient SBP response after the intervention (24); (ii) stenting CoA earlier in life have a protective effect against late hypertension, resulting in a more significant SBP decrease during follow-up (23). Since no significant difference in SBP was observed between studies with different follow-up durations, the former explanation may have contributed more to our observation.

Our study chronicles the results over time. Most stents used were CCP, followed by Advanta and BeGraft stents. The disparity in the number of implantations across these groups must be noted. Caution is advised when comparing the 288 CCP stents to the 23 BeGraft stents, as the coverage quality of CCP stents is widely recognized as suboptimal. Despite their longevity and extensive use, they may no longer be preferred. In addition, more aortic complications were observed in studies with follow-up durations of ≤12 months. A potential explanation may be that most studies with <12 months of follow-up were preliminary, retrospective reports without large sample sizes. The higher incidence of complications may be attributed to the learning curve. In the subgroup analysis, the incidence of re-coarctation was higher after Advanta stent implantation, potentially due to the study by Bruckheimer et al., which reported long-term follow-up of up to 5 years (19). The incidence of re-coarctation is supposed to be longer with prolonged follow-up durations.

## Conclusion

CBS implantation in treating CoA is a safe and effective treatment. All current commercially available CBSs significantly reduce trans-coarctation PG, increase CoA diameter, and reduce SBP during follow-up. The incidence of complications and re-coarctation is acceptable. Subgroup analysis showed significantly more access site complications following BeGraft stent implantation. For patients younger than 20 years, CBSs also reduced SBP more significantly during follow-up. However, this meta-analysis is based on single-arm data from highly heterogeneous studies. Therefore, studies with larger cohort sizes, longer follow-up durations, and comparative data are needed to further validate the efficacy of CBSs in treating CoA. The overall conclusion of this meta-analysis, as well as the take-home message, is that covered stents should be considered the procedure of choice in most consecutive unselected cases of coarctation.

## Data Availability

The original contributions presented in the study are included in the article/Supplementary Material, further inquiries can be directed to the corresponding authors.
