# Supplementary material for: Endovascular treatment of aortic coarctation using covered balloon-expandable stents—a systematic review and meta-analysis
Source: Front Cardiovasc Med. 2024 Oct 17;11:1439458. doi: 10.3389/fcvm.2024.1439458 (PMC11524840; doi:10.3389/fcvm.2024.1439458)
Supplement: Supplementary file 1 [file Table1.pdf]

	Tzifa 2006	Butera 2007	Bruckheimer 2009	Tanous 2010	Chang 2012	Ohno 2013	Sohrabi 2014	Promphan 2020	Sasikumar 2020	Stassen 2021	Bruckheimer 2021	Yilmazer 2021
Were there clear criteria for inclusion in the case series?	Yes	Yes	Yes	Unclear	Yes	Yes	Yes	Yes	Yes	Yes	Yes	Unclear
Was the condition measured in a standard, reliable way for all participants included in the case series?	Yes	Yes	Yes	Yes	Yes	Yes	Yes	Yes	Yes	Yes	Yes	Yes
Were valid methods used for identification of the condition for all participants included in the case series?	Yes	Yes	Yes	Yes	Yes	Yes	Yes	Yes	Yes	Yes	Yes	Yes
Did the case series have consecutive inclusion of participants?	Yes	Yes	Yes	Unclear	Yes	Yes	Yes	Unclear	Yes	Yes	Yes	Yes
Did the case series have complete inclusion of participants?	Yes	Yes	Unclear	Yes	Yes	Yes	Unclear	Unclear	Unclear	Unclear	Unclear	Unclear
Was there clear reporting of the demographics of the participants in the study?	Yes	Yes	Yes	Yes	Yes	Yes	Yes	Yes	Yes	Yes	Yes	Yes
Was there clear reporting of clinical information of the participants?	Yes	Yes	Yes	Yes	Yes	Yes	Yes	Yes	Yes	Yes	Yes	Yes
Were the outcomes or follow up results of cases clearly reported?	Yes	Yes	Yes	Yes	Yes	Yes	Yes	Yes	Yes	Yes	Yes	Yes
Was there clear reporting of the presenting sites(s)/clinic(s) demographic information?	Unclear	Unclear	Unclear	Unclear	Unclear	Unclear	Unclear	Unclear	Unclear	Unclear	Unclear	Unclear
Was statistical analysis appropriate?	Yes	Yes	Yes	Yes	Yes	Yes	Yes	Yes	Yes	Yes	Yes	Yes

Supplementary Table S1 Quality assessment of included studies using the Joanna Briggs Institute (JBI) Checklist for Case Series.
