# Supplementary material for: Endovascular treatment of aortic coarctation using covered balloon-expandable stents—a systematic review and meta-analysis
Source: Front Cardiovasc Med. 2024 Oct 17;11:1439458. doi: 10.3389/fcvm.2024.1439458 (PMC11524840; doi:10.3389/fcvm.2024.1439458)
Supplement: Supplementary file 2 [file Datasheet1.pdf]

## 1. Search strategy in different databases

**PubMed**

((((((((((((((((((((stent) OR (stents)) OR (stenting)) OR (stented)) OR (percutaneous)) OR (catheter*)) OR (transcatheter)) OR (trans catheter)) OR (transluminal)) OR (endovascular)) OR (intravascular)) OR (transvascular)) OR (angioplast*)) OR (TEVAR)) OR (thoracic endovascular aortic repair)) OR (stent [MeSH])) OR (endovascular procedures[MeSH])) OR (Endovascular Aneurysm Repair[MeSH])) OR (endovascular stent grafting)) OR (endovascular aortic repairs)) AND ((((((coarctation*) OR (coarctated)) OR (coarctate)) OR (recoarctation*)) OR (re-coarctation*)) OR (aortic coarctation [MeSH]))

N=3056

**Embase**

(coarctation* OR coarctated OR coarctate OR recoarctation* OR 're coarctation*' OR 'aortic coarctation'/exp) AND ('endovascular surgery'/exp OR 'stent'/exp OR 'endovascular aneurysm repair'/exp OR stent OR stents OR stenting OR stented OR percutaneous OR catheter* OR transcatheter OR 'trans catheter' OR transluminal OR endovascular OR intravascular OR transvascular OR angioplast* OR tevar OR 'thoracic endovascular aortic repair'/exp OR 'thoracic endovascular aortic repair' OR 'endovascular stent grafting' OR 'endovascular aortic repair')

N =5540

**Cochrane librabry**

#1 (coarctation*):ti,ab,kw OR (coarctated):ti,ab,kw OR (coarctate):ti,ab,kw OR (recoarctation*):ti,ab,kw OR (aortic coarctation):ti,ab,kw

#2 (stent):ti,ab,kw OR (stents):ti,ab,kw OR (stenting):ti,ab,kw OR (stented):ti,ab,kw OR (percutaneous):ti,ab,kw

#3 (catheter*):ti,ab,kw OR (transcatheter):ti,ab,kw OR (trans catheter):ti,ab,kw OR (transluminal):ti,ab,kw OR (endovascular):ti,ab,kw

#4 (intravascular):ti,ab,kw OR (transvascular):ti,ab,kw OR (angioplast*):ti,ab,kw OR (TEVAR):ti,ab,kw OR (thoracic endovascular aortic repair):ti,ab,kw

#5 (endovascular stent grafting):ti,ab,kw OR (endovascular aortic repairs):ti,ab,kw OR (endovascular procedures):ti,ab,kw OR (Endovascular Aneurysm Repair):ti,ab,kw

#6 #2 or #3 or #4 or #5

#7 #1 and #6

N =42
